# Association of physical activity, sedentary behaviours and sleep duration with cardiovascular diseases and lipid profiles: a Mendelian randomization analysis

**DOI:** 10.1186/s12944-020-01257-z

**Published:** 2020-05-08

**Authors:** Zhenhuang Zhuang, Meng Gao, Ruotong Yang, Nan Li, Zhonghua Liu, Weihua Cao, Tao Huang

**Affiliations:** 1 Department of Epidemiology & Biostatistics, School of Public Health, Peking University, 38 Xueyuan Road, Bejing, 100191 China; 2 Institute of Reproductive and Child Health, Peking University/Key Laboratory of Reproductive Health, National Health Commission of the People’s Republic of China, Bejing, 100191 China; 3 Department of Statistics and Actuarial Science, The University of Hong Kong, Pok Fu Lam, Hong Kong; 4 Department of Global Health, School of Public Health, Peking University, Bejing, 100191 China; 5 Key Laboratory of Molecular Cardiovascular Sciences (Peking University), Ministry of Education, Bejing, 100191 China

**Keywords:** Physical activity, Cardiovascular diseases, Mendelian randomization, Causality

## Abstract

**Background:**

Observational studies have shown that moderate-to-vigorous physical activity (MVPA), vigorous physical activity (VPA), sedentary behaviours, and sleep duration were associated with cardiovascular diseases (CVDs) and lipid levels. However, whether such observations reflect causality remain largely unknown. We aimed to investigate the causal associations of physical activity, sedentary behaviours, and sleep duration with coronary artery disease (CAD), myocardial infarction (MI), stroke and lipid levels.

**Methods:**

We conducted a Mendelian randomization (MR) study using genetic variants as instruments which are associated with physical activity, sedentary behaviours, and sleep duration to examine the causal effects on CVDs and lipid levels. This study included analyses of 4 potentially modifiable factors and 7 outcomes. Thus, the threshold of statistical significance is *P* = 1.8 × 10^− 3^ (0.05/4 × 7) after Bonferroni correction.

**Results:**

In the present study, there was suggestive evidence for associations of genetically predicted VPA with CAD (odds ratio, 0.65; 95% confidence intervals, 0.47–0.90; *P* = 0.009) and MI (0.74; 0.59–0.93; *P* = 0.010). However, genetically predicted VPA, MVPA, sleep duration and sedentary behaviours did not show significant associations with stroke and any lipid levels.

**Conclusions:**

Our findings from the MR approach provided suggestive evidence that vigorous exercise decreased risk of CAD and MI, but not stroke. However, there was no evidence to support causal associations of MVPA,sleep duration or sedentary behaviours with cardiovascular risk and lipid levels.

**Translational perspective:**

The findings of this study did not point out specific recommendations on increasing physical activity required to deliver significant health benefits. Nevertheless, the findings allowed clinicians and public health practitioners to provide advice about increasing the total amount of excising time by demonstrating that such advice can be effective. Reliable assessment of the association of physical activity levels with different subtypes of CVDs is needed to provide the basis for a comprehensive clinical approach on CVDs prevention, which can be achieved through lifestyle interventions in addition to drug therapy.

## Background

Cardiovascular diseases (CVDs) have become serious public health issues, which are the leading causes of death globally [1]. Etiology of CVDs is complex, including genetics and environmental factors such as diet and lifestyle [2–4]. A composite of light PA, sufficient sleep, and high sedentariness was responsible for the increased risk of CVDs according to a cross-sectional study [5]. Several observational studies have provided evidence for the significant association between CVDs and physical inactivity [6–8]. Importantly, a recent meta-analysis of 36 studies concluded that increased levels of physical activity was significantly associated with lower risk of CVDs [9]. Nevertheless, such observational studies were influenced by the possibility of confounding and reserve causation. In addition, epidemiological researches mainly rely on self-reported information, which may be inaccurate and subject to misclassification.

A recent community-based randomized controlled trial (RCT) suggested that physical activity had beneficial effects on adverse cardiovascular events in both the short- and medium-term [10]. Furthermore, there was a small benefit for reducing sedentary time to improve biomarkers of cardiometabolic risk in a cluster RCT [11]. Although RCTs have explored the effect of physical activity and sedentary behaviours on cardiovascular risk, the results are still inconclusive [10, 11]. Taken the limited duration of intervention trials into account, the information on potential long-term side effects may not be obtained, which also leads to bias in the results [12]. Therefore, the causal associations of physical activity, sedentary behaviours, and sleep duration with CVD remain uncertain.

The two-sample mendelian randomization (MR) is a widely used method for evaluating the casual relationships between risk factors and disease outcomes [13–16]. MR exploits the fact that alleles are randomly assigned from parents to offspring, which are unlikely to be affected by confounding factors. Moreover, issues of reverse causation are avoided because genotypes that are fixed at zygote formation could not be affected by diseases [17]. A recent MR study has shown that genetic instruments can be used to reveal causal relationships of physical activity with the risk of disease outcomes [18]. Therefore, such a method can be used in a large study design to uncover if and how lifestyle factors cause CVDs, under the premise that necessary assumptions are satisfied.

Therefore, in the present study, we used this MR approach to estimate the causal effect of physical activity, sedentary behaviours, and sleep duration on CVDs such as stroke, myocardial infarction (MI) and coronary artery disease (CAD). In complementary analysis, we investigated the role of four lifestyle factors on lipids including high density lipoprotein (HDL), low density lipoprotein (LDL), total cholesterol (TC) and triglycerides (TG).

## Methods

### Exposure and instrumental variables

We selected four lifestyle factors that have been shown to be strongly associated with CVDs in observational studies, including moderate-to-vigorous physical activity (MVPA), vigorous physical activity (VPA) [19–21], sedentary behaviours [22, 23], and sleep duration [24–26]. Then we searched PubMed and identified 3 publications reporting genome-wide association studies (GWAS) conducted for four lifestyle factors [27–29]. From these GWASs, 9 single nucleotide polymorphisms (SNPs) were identified with MVPA, 5 SNPs were identified with VPA, 4 SNPs were identified with sedentary behaviours and 7 SNPs were identified with sleep duration, showing strong association for genome-wide significance (*P* < 5 × 10^− 8^) with four lifestyle factors respectively. Besides, we chose the genetic variant with the lowest *P* value for association with each lifestyle factor if genetic variants are in linkage disequilibrium (LD). We included summarized statistics (effect size estimates and their standard errors) on four lifestyle factors including MVPA, VPA, sedentary behaviours and sleep duration from published GWAS (Table 1). The SNPs significantly associated with the lifestyle factors in our study were used as instrumental variables (IVs) for lifestyle factors.

**Table 1:** Genome-wide association studies and detailed information of single nucleotide polymorphisms used as instrumental variable in the Mendelian randomization analyses of lifestyle factors in relation to cardiovascular diseases

Traits	SNP	Ethnics	Chr	Position	Nearest gene	EA	EAF	BETA	SE	Sample size	P value	Study	Reference	Journal
Sedentary behaviours	rs26579	Europeans	5	87,985,295	MEF2C-AS2	G	0.415	0.028	0.005	91,105	2.60 × 10^− 09^	UK Biobank	Aiden Doherty et al.,2018^28^	Nat Commun
Sedentary behaviours	rs25966	Europeans	5	106,822,908	EFNA5	G	0.531	0.028	0.005	91,105	3.00 × 10^− 09^	UK Biobank	Aiden Doherty et al.,2018^28^	Nat Commun
Sedentary behaviours	rs6801032	Europeans	3	68,527,135	LOC105377146	A	0.259	0.031	0.005	91,105	3.10 × 10^−09^	UK Biobank	Aiden Doherty et al.,2018^28^	Nat Commun
Sedentary behaviours	rs7779206	Europeans	7	71,723,883	CALN1	A	0.558	0.028	0.005	91,105	4.20 × 10^−09^	UK Biobank	Aiden Doherty et al.,2018^28^	Nat Commun
MVPA	rs429358	Europeans	19	45,411,941	APOE	T	0.850	−0.019	0.003	377,234	7.30 × 10^−11^	UK Biobank	Klimentidis YC et al.,2018^27^	Int J Obes (Lond)
MVPA	rs7804463	Europeans	7	133,447,651	EXOC4	T	0.530	0.013	0.002	377,234	4.10 × 10^−10^	UK Biobank	Klimentidis YC et al.,2018^27^	Int J Obes (Lond)
MVPA	rs2854277	Europeans	6	32,628,084	HLA-DQB1	C	0.920	0.027	0.005	377,234	1.40 × 10^−08^	UK Biobank	Klimentidis YC et al.,2018^27^	Int J Obes (Lond)
MVPA	rs1379183	Europeans	7	50,237,784	C7orf72/SPATA48	C	0.410	−0.012	0.002	377,234	1.70 × 10^−08^	UK Biobank	Klimentidis YC et al.,2018^27^	Int J Obes (Lond)
MVPA	rs3094622	Europeans	6	30,327,952	RPP21	A	0.860	0.018	0.003	377,234	1.00 × 10^−08^	UK Biobank	Klimentidis YC et al.,2018^27^	Int J Obes (Lond)
MVPA	rs149943	Europeans	6	28,002,388	ZNF165	G	0.850	0.016	0.003	377,234	5.60 × 10^−08^	UK Biobank	Klimentidis YC et al.,2018^27^	Int J Obes (Lond)
MVPA	rs2035562	Europeans	3	85,056,521	CADM2	A	0.330	−0.014	0.002	377,234	1.00 × 10^−09^	UK Biobank	Klimentidis YC et al.,2018^27^	Int J Obes (Lond)
MVPA	rs7854466	Europeans	9	37,044,388	PAX5	T	0.560	−0.014	0.002	377,234	2.40 × 10^−11^	UK Biobank	Klimentidis YC et al.,2018^27^	Int J Obes (Lond)
MVPA	rs1043595	Europeans	7	128,410,012	CALU	G	0.720	0.013	0.002	377,234	4.20 × 10^−08^	UK Biobank	Klimentidis YC et al.,2018^27^	Int J Obes (Lond)
VPA	rs1248860	Europeans	3	85,015,779	CADM2	G	0.480	−0.051	0.007	261,055	5.30 × 10^−15^	UK Biobank	Klimentidis YC et al.,2018^27^	Int J Obes (Lond)
VPA	rs2764261	Europeans	6	108,927,842	FOXO3	A	0.370	0.030	0.005	261,055	5.30 × 10^−08^	UK Biobank	Klimentidis YC et al.,2018^27^	Int J Obes (Lond)
VPA	rs13243553	Europeans	7	133,506,955	EXOC4	G	0.610	0.039	0.007	261,055	2.40 × 10^−09^	UK Biobank	Klimentidis YC et al.,2018^27^	Int J Obes (Lond)
VPA	rs3781411	Europeans	10	126,715,436	CTBP2	C	0.880	0.058	0.009	261,055	1.00 × 10^−10^	UK Biobank	Klimentidis YC et al.,2018^27^	Int J Obes (Lond)
VPA	rs328902	Europeans	7	35,020,843	DPY19L1	C	0.690	−0.041	0.006	261,055	1.30 × 10^−10^	UK Biobank	Klimentidis YC et al.,2018^27^	Int J Obes (Lond)
Sleep duration	rs1191685	Europeans	2	113,811,454	PAX8	C	0.370	2.870	0.470	44,563	1.06 × 10^−09^	CHARGE	Daniel J. Gottlieb et al,2015^29^	Mol Psychiatry
Sleep duration	rs1823125	Europeans	2	113,806,882	PAX8	G	0.260	3.010	0.500	45,281	1.71 × 10^−08^	CHARGE	Daniel J. Gottlieb et al,2015^29^	Mol Psychiatry
Sleep duration	rs1807282	Europeans	2	113,826,506	PAX8	T	0.260	2.890	0.490	46,805	3.91 × 10^−09^	CHARGE	Daniel J. Gottlieb et al,2015^29^	Mol Psychiatry
Sleep duration	rs1964463	Europeans	2	113,785,491	PAX8	G	0.250	2.840	0.500	45,281	1.07 × 10^−08^	CHARGE	Daniel J. Gottlieb et al,2015^29^	Mol Psychiatry
Sleep duration	rs4587207	Europeans	6	30,874,924	IER3	G	0.800	−3.140	0.560	46,807	2.02 × 10^−08^	CHARGE	Daniel J. Gottlieb et al,2015^29^	Mol Psychiatry
Sleep duration	rs4248149	Europeans	6	30,875,606	IER3	C	0.800	−3.080	0.560	46,810	3.95 × 10^−08^	CHARGE	Daniel J. Gottlieb et al,2015^29^	Mol Psychiatry
Sleep duration	rs2394403	Europeans	6	30,875,848	IER3	T	0.800	−3.070	0.560	46,811	4.39 × 10^−08^	CHARGE	Daniel J. Gottlieb et al,2015^29^	Mol Psychiatry

*SNP* single nucleotide polymorphism; *Chr* chromosome; *EA* effect allele; *EAF* effect allele frequency; *SE* standard error; *MVPA* moderate-to-vigorous physical activity; *VPA* vigorous physical activity; *CHARGE* the Cohorts for Heart and Aging Research in Genomic Epidemiology

### Data sources

For disease outcomes, the summary statistics are extracted from the Coronary ARtery DIsease Genome wide Replication and Meta-analysis (CARDIoGRAM) plus the Coronary Artery Disease (C4D) Genetics (CARDIoGRAMplusC4D) consortium for CAD (60,801 cases and 123,504 controls), and MI (43,676 cases and 128,197 controls), respectively [30]; from the NINDS Stroke Genetics Network (SiGN) and International Stroke Genetics Consortium (ISGC) for stroke (37,792 cases and 397,209 controls) [31]. For lipid profiles, we extracted summary-level data from the Global Lipids Genetics Consortium (GLGC) consortium (*n* = 188,577) for lipid levels such as HDL, LDL, TC and TG [32]. Detailed information about summary data for the associations of the genetic variants with the lifestyle factors and CVDs and lipid levels are presented in Table 2 and **eTable 1–7**.

**Table 2:** Description of cardiovascular outcomes

Cardiometabolic factors	Consortium or study	Sample size^#^	Population	Year
Lipids	The Global Lipids Genetics	188,578	Trans-Ethnics	2013
Diseases				
CAD	CARDIoGRAM and CARDIoGRAMplusC4D	60,801/123,504	Trans-Ethnics	2015
MI	CARDIoGRAM and CARDIoGRAMplusC4D	43,676/128,197	Trans-Ethnics	2015
Stroke	SiGN and ISGC	37,792/397,209	Trans-Ethnics	2016

^#^Reports total sample size or case/controls;

Lipids include high density lipoprotein (mg/dL), low density lipoprotein (mg/dL), total cholesterol (mg/dL), triglycerides (mg/dL)

CAD: coronary artery disease; MI: myocardial infarction; CARDIoGRAMplusC4D: the Coronary ARtery DIsease Genome wide Replication and Meta-analysis (CARDIoGRAM) plus the Coronary Artery Disease (C4D) Genetics (CARDIoGRAMplusC4D) consortium; SiGN: the NINDS Stroke Genetics Network; ISGC: International Stroke Genetics Consortium

CAD cases status were defined broadly, including MI, acute coronary syndrome, chronic stable angina, or coronary artery stenosis greater than 50% [30]. Stroke cases were recruited between 1989 and 2012, aged 16 to 104 years, and subtypes of ischaemic stroke were recorded by centrally trained and certified investigators who used the web-based protocol, Causative Classification of Stroke (CCS) [31]. In addition, lipids GWAS evaluated the additive effects of each SNP on blood lipid levels after adjusting for age and sex, and individuals known to be on lipid levels lowering medications were excluded [32].

### Mendelian randomization analysis

**fig. 1:**
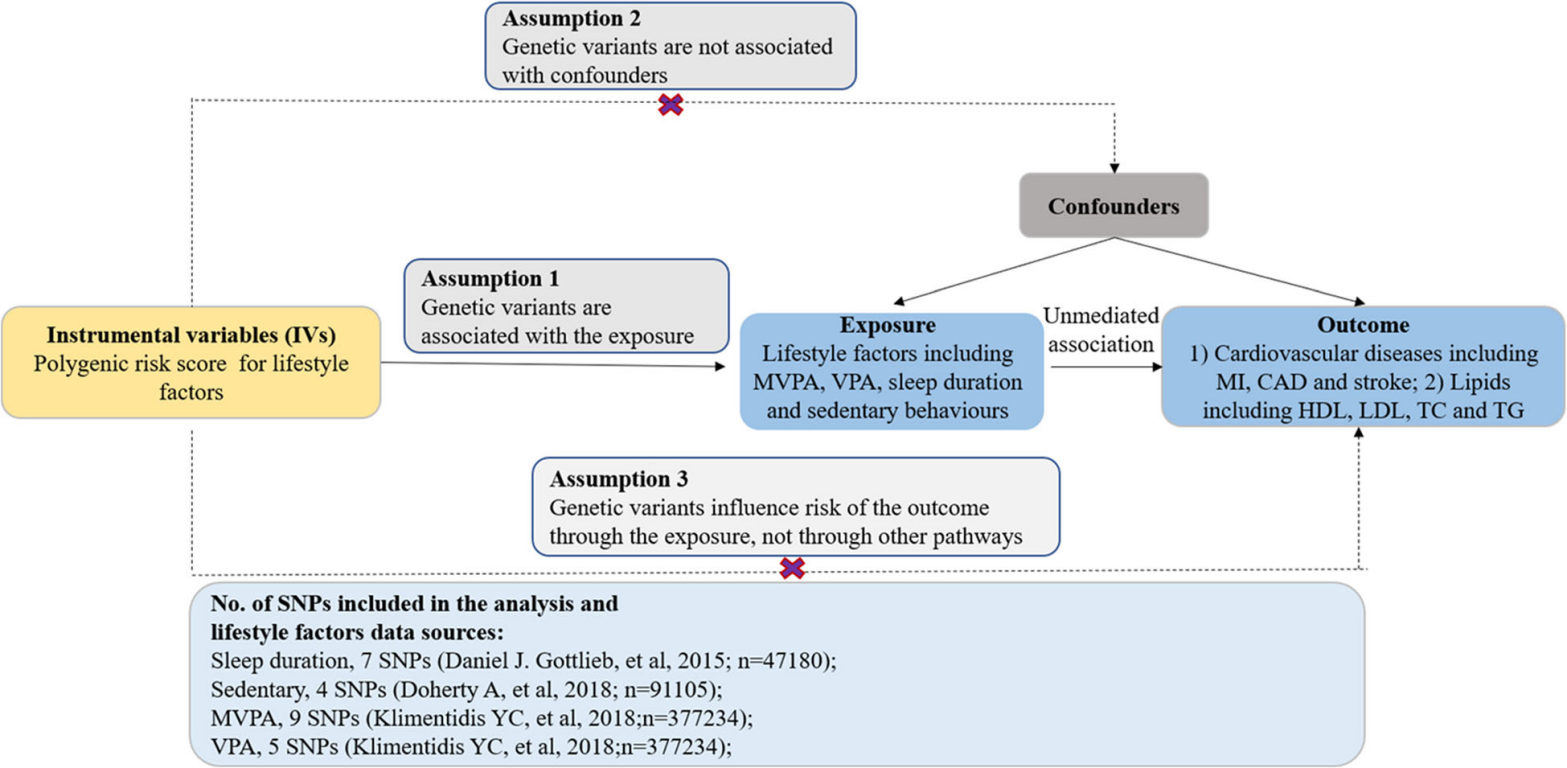
Assumptions of a Mendelian randomization analysis for lifestyle factors and risk of cardiovascular diseases. Broken lines represent potential pleiotropic or direct causal effects between variables that would violate Mendelian randomization assumptions. MVPA = moderate-to-vigorous physical activity, VPA = vigorous physical activity; CAD = coronary artery disease, MI = myocardial infarction

A two-sample MR method was used in the present study using summary level data with a beta-coefficient and the standard error from the regression of the lifestyle factors on the genotype and similar data for the regression of the disease outcomes on the genotype [12]. Recently, the two-sample MR method is widely applied because of the large amount of public data from the global GWAS collaboration group. To make genetic variant qualified as a valid instrument for causal inference, the MR approach we used must satisfy the following three core assumptions: genetic variants are associated with the exposure; genetic variants are not associated with confounders; genetic variants influence risk of the outcome only through the exposure, not through other pathways (Fig. 1).

We first harmonized the effect of exposure and outcome data sets containing combined information on SNPs, phenotype, effect allele, effect size, standard error for selected SNPs. In the main analyses, we calculated the odds ratio (OR) and 95% confidence intervals (CIs) for IVs by dividing the per-allele log-OR of CVDs by the per-allele difference in four lifestyle factors for each genetic variant respectively, using four different MR methods in which the conventional fixed effect inverse variance weighted method (IVW) is in a key position to get causal estimates. Besides, simple median method, weighted median method and MR-Egger regression method are performed as sensitivity analyses for lifestyle factors that are found to be significantly associated with CVDs. The weighted median method has a high tolerance for pleiotropy, which provides a consistent estimate if at least 50% of the weight comes from valid SNPs. To see if there is directional pleiotropy existing in the IVW estimates, the MR-Egger analysis was conducted to test whether there is evidence of the intercept parameter being different from zero. In the absence of directional pleiotropy, the IV estimates of each SNP should be distributed symmetrically near the point estimation, indicating that there is no systematic bias in the results. Heterogeneity in odds ratio was quantified using the I^2^ test.

The results of the present study are shown as OR (95% CIs) per genetically predicted increase in each lifestyle factor. We selected SNPs associated with self-reported levels of physical activity which were measured via a touchscreen questionnaire in the UK Biobank [27]. The measurement of MVPA like carrying light loads, cycling at normal pace was taking the sum of total minutes/week of MVPA multiplied by four and the total number of VPA minutes/week multiplied by eight, which were corresponding to metabolic equivalents. However, minutes/week of VPA such as fast cycling, aerobics, heavy lifting were divided into those meeting the 3 days/week of VPA at 25/mins per day vs. 0 days of VPA because the heritability of VPA has previously been shown to be high [27]. Sedentary was measured by self-reported duration of sedentary behaviours, which may occur in sitting, reclining or lying postures [28]. Furthermore, sleep duration was measured by self-reported usual hours of sleep at night [29]. It’s worth noting that the estimates represent the odds ratio per 1 unit higher log odds of the risk factor for the binary risk factors like VPA.

All analyses were performed with Stata version 14.2 (StataCorp, College Station, TX) and R version 3.5.3 (R foundation). Taken multiple testing into account, we applied Bonferroni correction in the genetic association analyses, thus the threshold of statistical significance is 0.05 / (3 outcomes × 4 factors) = 0.00417. *P* < 0.05 but above the Bonferroni corrected significance threshold was considered as suggestive evidence for a potential association.

## Results

### Coronary artery disease (CAD)

**Fig. 2:**
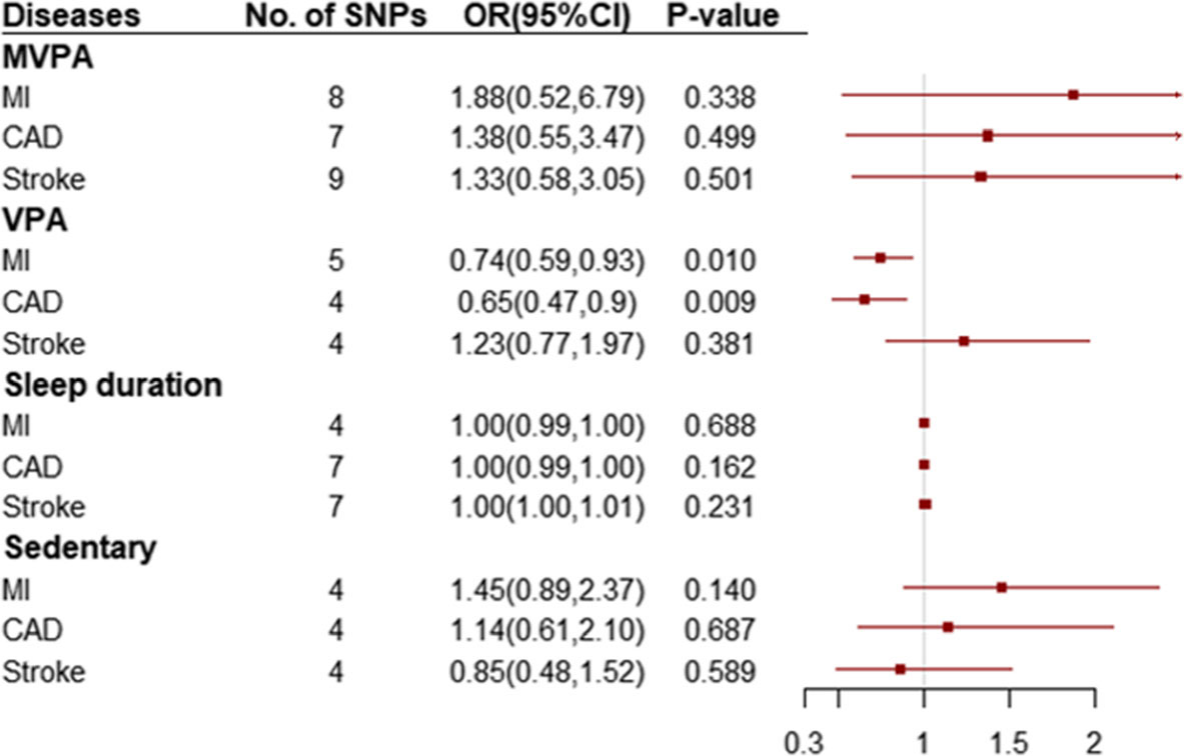
Odds ratio for association of genetically predicted lifestyle factors with cardiovascular diseases. OR: odds ratio; CI: confidence internal. OR (95% CI) means risk of cardiovascular diseases per 1 SD increase of continuous factors or per 1 unit log odds increase of binary factors. SNPs = single nucleotide polymorphisms; MVPA = moderate-to-vigorous physical activity, VPA = vigorous physical activity; MI = myocardial infarction, CAD = coronary artery disease

We found evidence of a potential protective effect of genetically predicted VPA on CAD (IVW OR, 0.65 for CAD per 1 unit higher log odds in VPA; 95% CI, 0.47–0.90; *P* = 0.009) (Fig. 2**and eFigure 1**); simple median and weighted median yielded similar pattern of effects; the results of the MR-Egger method confirmed the absence of evidence for directional pleiotropy (*P* = 0.258) **(eTable 8**). However, there was no significant association between CAD and genetically predicted MVPA (IVW OR, 1.38 for CAD per 1-SD higher in MVPA; 95% CI, 0.55–3.47; *P* = 0.499), genetically predicted sleep duration (IVW OR, 1.00 for CAD per 1-SD higher in sleep duration; 95% CI, 0.99–1.00; *P* = 0.162) or genetically predicted sedentary behaviours (IVW OR, 1.14 for CAD per 1-SD higher in sedentary behaviours; 95% CI, 0.61–2.10; *P* = 0.687), which provided insufficient data for alternative MR methods and sensitivity analyses (Fig. 2).

### Myocardial infarction (MI)

Our MR results suggested higher log odds of genetically predicted VPA was associated with lower risk of MI (IVW OR, 0.74 for MI per 1 unit higher log odds in VPA; 95% CI, 0.59–0.93; *P* = 0.010) (Fig. 2**and eFig. 2**); the simple median and the weighted median methods obtain consistent results with the IVW; there is no evidence of directional pleiotropy in the genetic instrument using the Egger intercept test (*P* = 0.560) **(eTable 8**). Nonetheless, we observed that genetically predicted MVPA (IVW OR, 1.88 for MI per 1-SD higher in MVPA; 95% CI, 0.52–6.79; *P* = 0.338), genetically predicted sleep duration (IVW OR, 1.00 for MI per 1-SD higher in sleep duration; 95% CI, 1.00–1.00; *P* = 0.688), and genetically predicted sedentary behaviours (IVW OR, 1.45 for MI per 1-SD higher in sedentary behaviours; 95% CI, 0.89–2.37; *P* = 0.140) were not significant associated with MI (Fig. 2).

### Stroke

There was no significant association to be found between four lifestyle factors and stroke, including genetically predicted MVPA (IVW OR, 1.33 for stroke per 1-SD higher in MVPA; 95% CI, 0.58–3.05; *P* = 0.501), genetically predicted sleep duration (IVW OR, 1.00 for stroke per 1-SD higher in sleep duration; 95% CI, 1.00–1.01; *P* = 0.231), genetically predicted VPA (IVW OR, 1.23 for stroke per 1 unit higher log odds in VPA; 95% CI, 0.77–1.97; *P* = 0.381 and genetically predicted sedentary behaviours (IVW OR, 0.85 for stroke per 1-SD higher in sedentary behaviours; 95% CI, 0.48–1.52; *P* = 0.589) (Figure 2).

### Lipids

**Fig. 3:**
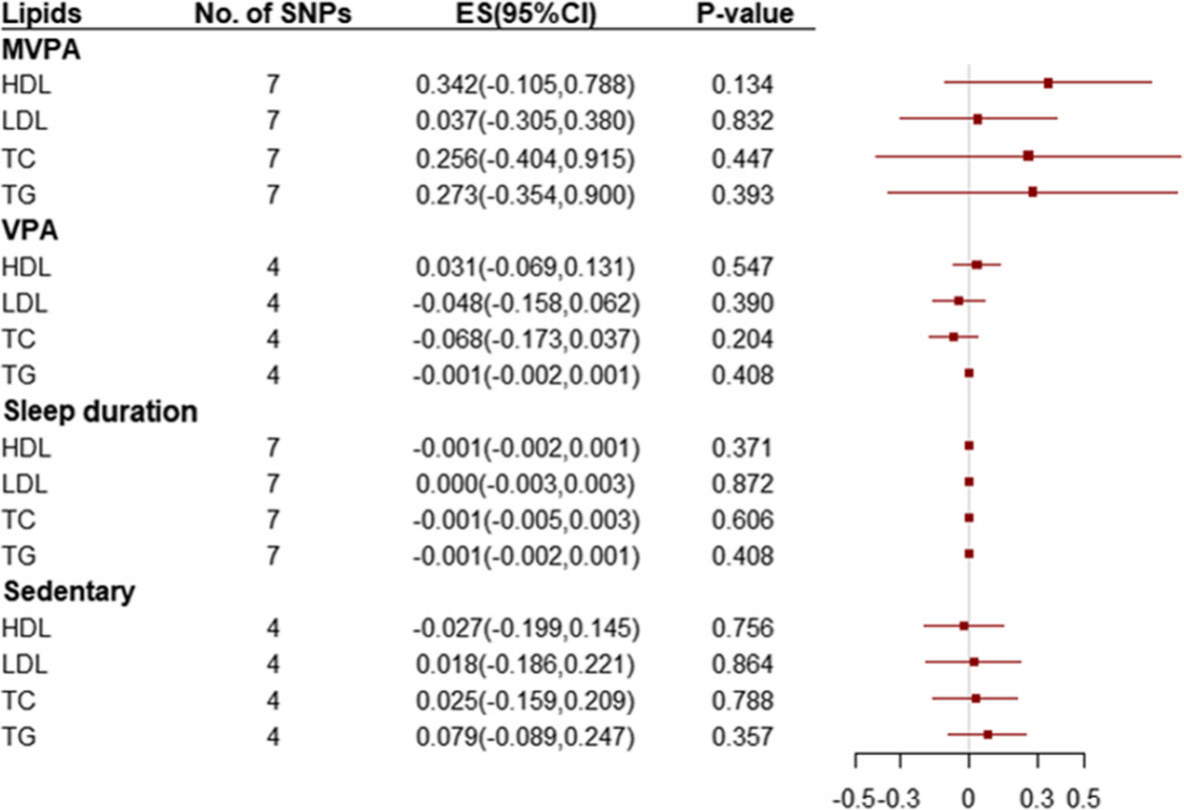
Odds ratio for association of genetically predicted lifestyle factors with lipids. ES: effect size; CI: confidence internal. ES (95% CI) means risk of changes in lipids per 1 SD increase of continuous factors or per 1 unit log odds increase of binary factors. SNPs = single nucleotide polymorphisms; MVPA = moderate-to-vigorous physical activity, VPA = vigorous physical activity; HDL = high density lipoprotein, LDL = low density lipoprotein, TC = total cholesterol, TG = triglycerides

We further examined the causal effects of the lifestyle factors on HDL, LDL, TC and TG, and we did not find that lifestyle factors SNPs were associated with any lipids (Fig. 3). Results were similar in sensitivity analyses that used the simple median and weighted median methods. However, directional pleiotropy existed in the associations of MVPA or sleep duration with almost all lipids (P<0.05), which may influence the results. (**eTable 9**).

## Discussion

In the present study, using the MR approach, we for the first time investigated whether the previously observed significant associations of physical activity, sedentary behaviours and sleep duration with risk of CVDs were causal. The MR analysis supported the hypothesis that vigorous exercise may be an effective prevention strategy for CAD and MI. However, there was no evidence using this method and these SNPs to support the associations of MVPA, sedentary behaviours or sleep duration with cardiovascular outcomes and lipids.

Several observational studies have investigated the association of self-reported physical activity levels with incidence of CVDs. According to the PURE study, a prospective cohort study in 130 000 people from 17 high-income, middle-income, and low-income countries, higher recreational and non-recreational physical activity was associated with a lower risk of mortality and CVD events [20]. A population-based prospective cohort study, which included 487,334 adults in 10 (5 urban, 5 rural) areas across China, suggested that total physical activity was inversely associated with the risk of major vascular events among Chinese adults [33]. A protective effect of physical activity on CVDs has also been observed in a smaller cohort study with median follow-up of 6.7 years [34]. Some epidemiology studies, mostly of RCTs, indicated that physical activity interventions was beneficial to improving cardiovascular health [10, 35]. To our best knowledge, the present study may be the first time to assess physical activity as a causal risk factor for CVDs including CAD, MI and stroke using the MR design with summarized data from GWAS with up to about 377,000 individuals [27]. The association appeared to be driven by VPA, whereas no association was observed with MVPA, which meant that vigorous activity levels seems to be more important than total activity time on high cardiovascular fitness [36].

We are not aware of any previous MR study assessing the association of generally predicted physical activity with CAD and MI. Our results of the potential relationship between physical activity and CAD or MI were consistent with a prospective population-based Rotterdam Study among 5901 elderly participants showing that specific physical activity types provided beneficial effects on risk of CAD [37]. Likewise, higher levels of leisure-time physical activity had a strong, graded, inverse association with the risk of acute myocardial infarction in a cohort study of 5-year follow-up [38]. In addition, a case-control study suggested that sports-related physical activity is associated with a lower risk of onset of AMI than inactivity in Chinese CAD patients [39].

Nevertheless, we found no significant association of physical activity with stroke, different from conclusions of previous observational studies that suggested physical activity reduced the risk of stroke [40, 41]. But this MR study confirmed the findings from a Japanese prospective study showing that excessive vigorous-intensity activities might not be beneficial or even disadvantageous for prevention of hemorrhagic stroke for Japanese people [42]. Likewise, there was few significant effect of physical activity on lipid profiles among groups in intervention studies, which was consistent with our null finding [43, 44]. Further investigations are warranted to elucidate the potential association between physical activity and different stroke subtypes or lipids.

Furthermore, in the present MR analysis, there was no evidence of association of genetically predicted sleep duration, MVPA and sedentary behaviours with CVDs and lipids. Our results did not support previous finds from some observational studies, which showed that MVPA favorably reduced cardiometabolic risk [21] and sedentary behaviours and long sleep duration caused prejudice to cardiometabolic health [22–24] respectively. However, results from these epidemiology studies were inconsistent [25, 45, 46]. Our null findings suggested that the previously observational association between the lifestyle factors and cardiometabolic disease in earlier observational studies might be false positive which mainly attributed to reverse causation or confounding. The present analysis, using the genetic variants strongly associated with lifestyle factors explain a larger part of variance, shows the true null association. We cannot exclude, however, the possibility that such association may have been diluted because of the potential pleiotropy due to lots of variants. Therefore, further large intervention trial is needed to explore the effect of sleep duration, MVPA and sedentary behaviours on cardiovascular risk.

Accumulating evidence have suggested that regular physical activity has known health benefits and is associated with reduced risk of CVDs. According to previous study, VPA is defined as physical activities that make participants sweat or breathe hard such as fast cycling, aerobics and heavy lifting, without precise index measurements [27]. Increasing VPA can lower blood pressure, blood glucose and weight and improve lipid profile, which prevent primary and secondary cardiovascular events in both young and older adults [47]. However, the mechanistic pathways underlying the cardiovascular benefits of VPA are still not fully understood. One benefit of VPA is that the accumulated hemodynamic stimulation during aerobic exercise can induce anti-atherosclerotic adaptation of vascular function and structure by releasing nitric oxide and myokines in ductal arteries that expand with movement, independent from other risk factors of CVDs [48]. Another interpretation is that VPA can also increase myocardial perfusion in the coronary collateralization, which not only improves blood flow to the heart but also protects against serious arrhythmias by cardiac autonomic balance improvement [48]. Increasing physical activity has been considered a simple, widely applicable, low cost global strategy that could reduce the risk of onset and deaths of CVDs. Recognizing the biological processes by which VPA affect the development of CVDs may help to understand the potential mechanisms.

Several strengths of this study merit consideration. First, we for the first time systematically assessed the causal effects of physical activity, sedentary behaviours and sleep duration in the development of CVDs using a MR approach. The MR analysis provided a better mean of generating a relatively less confounded estimates of causal relationships inferred without influenced by reverse causal effects or confounding. Second, in addition to traditional IVW method, we also adopted simple median method, weighted median method and MR-Egger method as sensitivity analysis to ensure the consistency of causal estimation, suggesting robustness of our findings. Third, besides this design technique, strengths of this study also included that summary statistics were collected from GWAS which included larger sample size than epidemiological studies, suggesting a higher statistical power for reliable causal estimation.

However, MR studies still have potential limitations although core assumptions have been met. First, genetic variants identified in GWAS often have small phenotypic effects, resulting in potentially weak instruments bias which depends on the strength of the genetic instruments through the F statistic. Second, population stratification is another limitation, which refers to the existence of the differences in allele frequencies and disease prevalence in different ethnic groups. Third, we assumed that the associations between lifestyle factors and CVDs are linear, which may not be consistent with the fact. Hence, the role of lifestyle factors including physical activity, sleep duration and sedentary behaviours in the development of CVDs needs further investigation. Finally, MR studies require large sample sizes to ensure enough power, which is not easy to calculate [49].

In conclusion, our study provided evidence to support the protective effect of vigorous exercise on CAD and MI, but not stroke, suggesting that aerobic exercise was a powerful approach for prevention of cardiovascular risk. However, larger intervention studies are needed to further explore the causal associations of physical activity, sedentary behaviours, and sleep duration with CVDs and lipid profiles.

## Supplementary information

**Additional file 1.** Online supplemental materials.

## Abbreviations

CVDs: Cardiovascular diseases
CAD: Coronary artery disease
CIs: Confidence intervals
GWAS: Genome-wide association studies
HDL: High density lipoprotein
IVW: Inverse variance weighted
LDL: Low density lipoprotein
MVPA: Moderate-to-vigorous physical activity
MI: Myocardial infarction
MR: Mendelian randomization
OR: Odds ratio
RCT: Randomized controlled trial
SNPs: Single nucleotide polymorphisms
TC: Total cholesterol
TG: Triglycerides
VPA: Vigorous physical activity
