# Supplementary material for: Association of physical activity, sedentary behaviours and sleep duration with cardiovascular diseases and lipid profiles: a Mendelian randomization analysis
Source: Lipids Health Dis. 2020 May 8;19:86. doi: 10.1186/s12944-020-01257-z (PMC7206776; doi:10.1186/s12944-020-01257-z)
Supplement: Supplementary file 1 — Additional file 1. Online supplemental materials. [file 12944_2020_1257_MOESM1_ESM.docx]

**Online supplemental materials**

**eFigure 1.** Association between genetically predicted vigorous physical activity and coronary artery disease.

**eFigure 2.** Association between genetically predicted vigorous physical activity and myocardial infarction.

**eTable 1.** Summary statistics for the genetic variants associated with the lifestyle factors investigated for an association with coronary artery disease in the present Mendelian randomization study.

**eTable 2.** Summary statistics for the genetic variants associated with the lifestyle factors investigated for an association with myocardial infarction in the present Mendelian randomization study.

**eTable 3.** Summary statistics for the genetic variants associated with the lifestyle factors investigated for an association with stroke in the present Mendelian randomization study.

**eTable 4.** Summary statistics for the genetic variants associated with the lifestyle factors investigated for an association with high density lipoprotein in the present Mendelian randomization study.

**eTable 5.** Summary statistics for the genetic variants associated with the lifestyle factors investigated for an association with low density lipoprotein in the present Mendelian randomization study.

**eTable 6.** Summary statistics for the genetic variants associated with the lifestyle factors investigated for an association with total cholesterol in the present Mendelian randomization study.

**eTable 7.** Summary statistics for the genetic variants associated with the lifestyle factors investigated for an association with triglycerides in the present Mendelian randomization study.

**eTable 8.** Associations between lifestyle factors (generally predicted per 1 SD increase of continuous factors or per 1 unit log odds increase of binary factors) and cardiovascular diseases in sensitivity analyses.

**eTable 9.** Associations between lifestyle factors (generally predicted per 1 SD increase of continuous factors or per 1 unit log odds increase of binary factors) and lipids in sensitivity analyses.

**eFigure 1.** **Association between genetically predicted vigorous physical activity and coronary artery disease.**

**
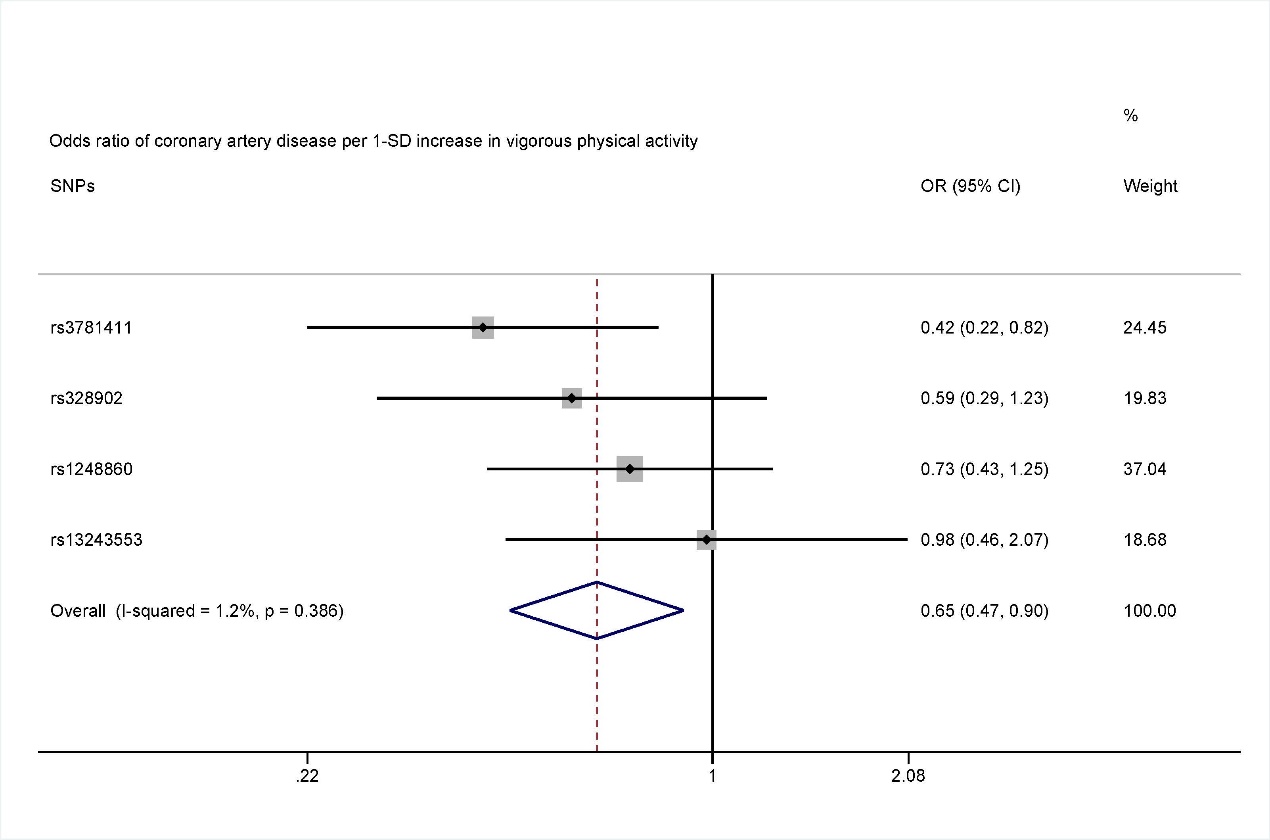
**

Squares represent the odds ratio of coronary artery disease per genetically predicted one unit increase in log-odds of vigorous physical activity; horizontal lines represent 95% confidence intervals (CIs); diamond represent the overall odds ratio with its 95% CI. SNP = single nucleotide polymorphism.

**eFigure 2.** **Association between genetically predicted vigorous physical activity and myocardial infarction.**

**
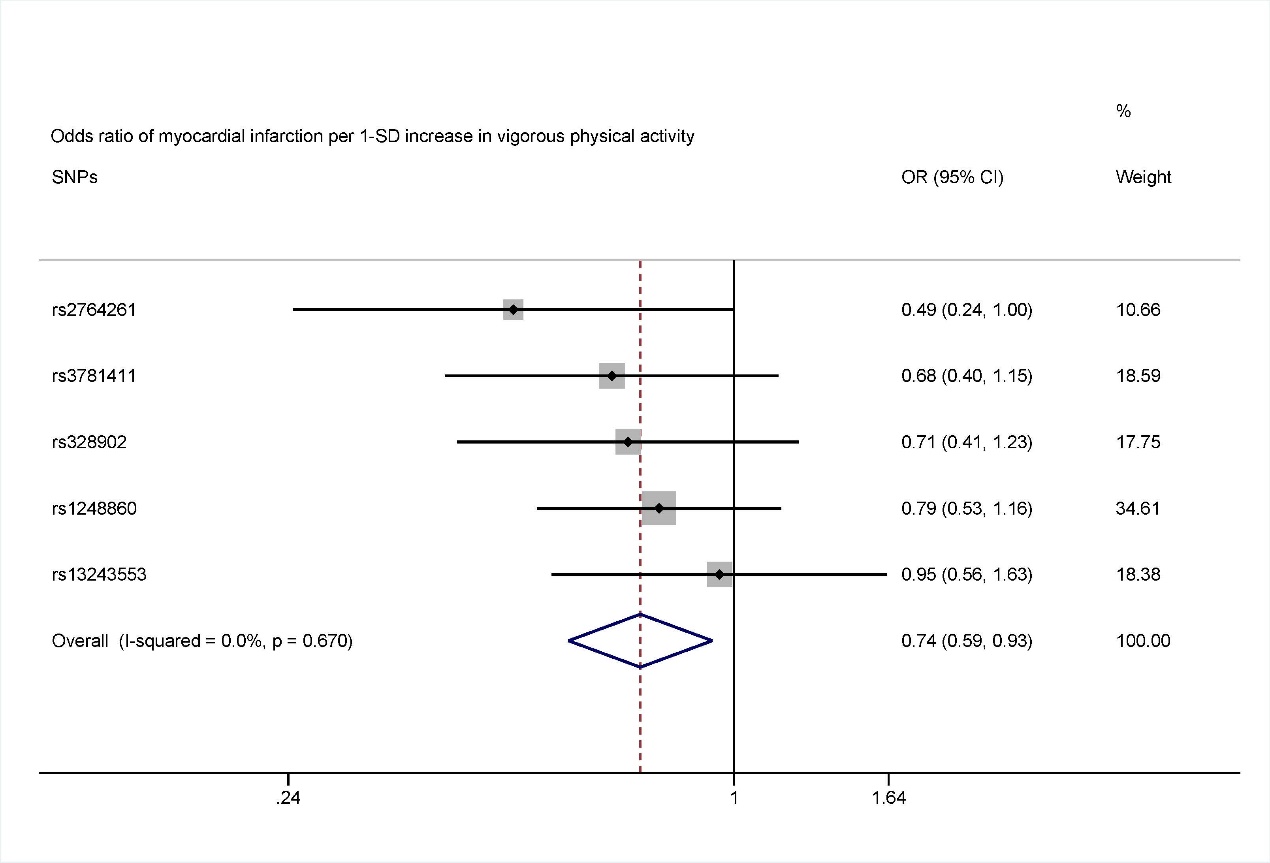
**

Squares represent the odds ratio of myocardial infarction per genetically predicted one unit increase in log-odds of vigorous physical activity; horizontal lines represent 95% confidence intervals (CIs); diamond represent the overall odds ratio with its 95% CI. SNP = single nucleotide polymorphism.

**eTable 1. Summary statistics for the genetic variants associated with the lifestyle factors investigated for an association with coronary artery disease in the present Mendelian randomization study.**

|  |  |  |  | **Lifestyle factor** | |  | **Coronary artery disease result** | | | |
| --- | --- | --- | --- | --- | --- | --- | --- | --- | --- | --- |
| **Lifestyle factor** | **SNP** | **Chr** | **EA** | **β** | **SE** |  | | **β** | **SE** | **P value** |
| MVPA | rs1043595 | 7 | G | 0.013 | 0.002 |  | | 0.0107 | 0.0174 | 0.5405 |
| MVPA | rs1379183 | 7 | A | 0.012 | 0.002 |  | | 0.0258 | 0.0147 | 0.0789 |
| MVPA | rs149943 | 6 | G | 0.016 | 0.003 |  | | 0.0039 | 0.0232 | 0.8654 |
| MVPA | rs2035562 | 3 | G | 0.014 | 0.002 |  | | -0.0213 | 0.0151 | 0.1587 |
| MVPA | rs3094622 | 6 | A | 0.018 | 0.003 |  | | 0.0146 | 0.0247 | 0.5544 |
| MVPA | rs7804463 | 7 | T | 0.013 | 0.002 |  | | 0.0141 | 0.0141 | 0.3174 |
| MVPA | rs7854466 | 9 | G | 0.014 | 0.002 |  | | -0.007 | 0.0141 | 0.6207 |
| Sedentary | rs25966 | 5 | G | 0.028 | 0.005 |  | | -0.015 | 0.014 | 0.2829 |
| Sedentary | rs26579 | 5 | G | 0.028 | 0.005 |  | | 0.019 | 0.0168 | 0.2574 |
| Sedentary | rs6801032 | 3 | A | 0.031 | 0.005 |  | | -0.0061 | 0.0162 | 0.7089 |
| Sedentary | rs7779206 | 7 | A | 0.028 | 0.005 |  | | 0.0196 | 0.0139 | 0.1605 |
| Sleep | rs1191685 | 2 | C | 2.870 | 0.470 |  | | 0.0055 | 0.0219 | 0.8005 |
| Sleep | rs1807282 | 2 | T | 2.890 | 0.490 |  | | -0.0267 | 0.0166 | 0.1084 |
| Sleep | rs1823125 | 2 | G | 3.010 | 0.500 |  | | -0.0294 | 0.017 | 0.0843 |
| Sleep | rs1964463 | 2 | G | 2.840 | 0.500 |  | | -0.0181 | 0.0163 | 0.2679 |
| Sleep | rs2394403 | 6 | C | 3.070 | 0.560 |  | | 0.006 | 0.0194 | 0.7588 |
| Sleep | rs4248149 | 6 | T | 3.080 | 0.560 |  | | 0.0065 | 0.0194 | 0.7354 |
| Sleep | rs4587207 | 6 | A | 3.140 | 0.560 |  | | 0.0044 | 0.0192 | 0.8165 |
| VPA | rs1248860 | 3 | A | 0.051 | 0.007 |  | | -0.0158 | 0.0139 | 0.2562 |
| VPA | rs13243553 | 7 | G | 0.039 | 0.007 |  | | -0.0009 | 0.015 | 0.9534 |
| VPA | rs328902 | 7 | T | 0.041 | 0.006 |  | | -0.0215 | 0.0152 | 0.1572 |
| VPA | rs3781411 | 10 | C | 0.058 | 0.009 |  | | -0.05 | 0.0195 | 0.0104 |

SNP = single nucleotide polymorphism; Chr = chromosome; EA = effect allele; SE = standard error; MVPA = moderate-to-vigorous physical activity; VPA = vigorous physical activity. *Effect size estimates (β) were obtained from published genome-wide association studies of four lifestyle factors and coronary artery disease and represent one unit change in the lifestyle factors per additional copy of the effect allele.

**eTable 2. Summary statistics for the genetic variants associated with the lifestyle factors investigated for an association with myocardial infarction in the present Mendelian randomization study.**

|  |  |  |  | **Lifestyle factor** | |  | **Myocardial infarction result** | | |
| --- | --- | --- | --- | --- | --- | --- | --- | --- | --- |
| **Lifestyle factor** | **SNP** | **Chr** | **EA** | **β** | **SE** |  | **β** | **SE** | **P value** |
| MVPA | rs1043595 | 7 | G | 0.013 | 0.002 |  | 0.0081 | 0.0133 | 0.5441 |
| MVPA | rs1379183 | 7 | A | 0.012 | 0.002 |  | 0.0060 | 0.0105 | 0.5685 |
| MVPA | rs149943 | 6 | G | 0.016 | 0.003 |  | 0.0128 | 0.0162 | 0.4295 |
| MVPA | rs2035562 | 3 | G | 0.014 | 0.002 |  | -0.0053 | 0.0108 | 0.6275 |
| MVPA | rs2854277 | 6 | C | 0.027 | 0.005 |  | 0.0190 | 0.0207 | 0.3572 |
| MVPA | rs429358 | 19 | C | 0.019 | 0.003 |  | 0.0962 | 0.0169 | 1.3682 |
| MVPA | rs7804463 | 7 | T | 0.013 | 0.002 |  | 0.0049 | 0.0105 | 0.6392 |
| MVPA | rs7854466 | 9 | G | 0.014 | 0.002 |  | -0.0194 | 0.0104 | 0.0632 |
| Sedentary | rs25966 | 5 | G | 0.028 | 0.005 |  | 0.0040 | 0.0106 | 0.7088 |
| Sedentary | rs26579 | 5 | G | 0.028 | 0.005 |  | -0.0037 | 0.0105 | 0.7258 |
| Sedentary | rs6801032 | 3 | A | 0.031 | 0.005 |  | 0.0129 | 0.0116 | 0.2669 |
| Sedentary | rs7779206 | 7 | A | 0.028 | 0.005 |  | 0.0290 | 0.0104 | 0.0052 |
| Sleep | rs1191685 | 2 | C | 2.870 | 0.470 |  | -0.0165 | 0.0103 | 0.1088 |
| Sleep | rs1807282 | 2 | T | 2.890 | 0.490 |  | 0.0073 | 0.0125 | 0.5589 |
| Sleep | rs1823125 | 2 | G | 3.010 | 0.500 |  | 0.0054 | 0.0121 | 0.6535 |
| Sleep | rs1964463 | 2 | G | 2.840 | 0.500 |  | 0.0000 | 0.0120 | 0.9996 |
| VPA | rs1248860 | 3 | A | 0.051 | 0.007 |  | -0.0123 | 0.0102 | 0.2287 |
| VPA | rs13243553 | 7 | G | 0.039 | 0.007 |  | -0.0019 | 0.0107 | 0.8624 |
| VPA | rs2764261 | 6 | A | 0.030 | 0.005 |  | -0.0209 | 0.0106 | 0.0491 |
| VPA | rs328902 | 7 | T | 0.041 | 0.006 |  | -0.0139 | 0.0114 | 0.2218 |
| VPA | rs3781411 | 10 | C | 0.058 | 0.009 |  | -0.0228 | 0.0159 | 0.1506 |

SNP = single nucleotide polymorphism; Chr = chromosome; EA = effect allele; SE = standard error; MVPA = moderate-to-vigorous physical activity; VPA = vigorous physical activity. *Effect size estimates (β) were obtained from published genome-wide association studies of four lifestyle factors and myocardial infarction and represent one unit change in the lifestyle factors per additional copy of the effect allele.

**eTable 3.** **Summary statistics for the genetic variants associated with the lifestyle factors investigated for an association with stroke in the present Mendelian randomization study.**

|  |  |  |  | **Lifestyle factor** | |  | **Stroke result** | | |
| --- | --- | --- | --- | --- | --- | --- | --- | --- | --- |
| **Lifestyle factor** | **SNP** | **Chr** | **EA** | **β** | **SE** |  | **β** | **SE** | **P value** |
| MVPA | rs1043595 | 7 | G | 0.013 | 0.002 |  | 0.0032 | 0.0181 | 0.8607 |
| MVPA | rs1379183 | 7 | A | 0.012 | 0.002 |  | 0.0070 | 0.0164 | 0.6719 |
| MVPA | rs149943 | 6 | G | 0.016 | 0.003 |  | -0.0165 | 0.0246 | 0.5040 |
| MVPA | rs2035562 | 3 | G | 0.014 | 0.002 |  | -0.0027 | 0.0172 | 0.8764 |
| MVPA | rs2854277 | 6 | C | 0.027 | 0.005 |  | 0.0177 | 0.0269 | 0.5100 |
| MVPA | rs3094622 | 6 | A | 0.018 | 0.003 |  | 0.0100 | 0.0276 | 0.7161 |
| MVPA | rs429358 | 19 | C | 0.019 | 0.003 |  | -0.0385 | 0.0242 | 0.1111 |
| MVPA | rs7804463 | 7 | T | 0.013 | 0.002 |  | 0.0216 | 0.0162 | 0.1832 |
| MVPA | rs7854466 | 9 | G | 0.014 | 0.002 |  | 0.0189 | 0.0163 | 0.2470 |
| Sedentary | rs25966 | 5 | G | 0.028 | 0.005 |  | 0.0015 | 0.0163 | 0.9252 |
| Sedentary | rs26579 | 5 | G | 0.028 | 0.005 |  | -0.0153 | 0.0165 | 0.3531 |
| Sedentary | rs6801032 | 3 | A | 0.031 | 0.005 |  | -0.0120 | 0.0185 | 0.5173 |
| Sedentary | rs7779206 | 7 | A | 0.028 | 0.005 |  | 0.0062 | 0.0162 | 0.7025 |
| Sleep | rs1191685 | 2 | C | 2.870 | 0.470 |  | -0.0040 | 0.0164 | 0.8083 |
| Sleep | rs1807282 | 2 | T | 2.890 | 0.490 |  | -0.0034 | 0.0198 | 0.8630 |
| Sleep | rs1823125 | 2 | G | 3.010 | 0.500 |  | -0.0024 | 0.0195 | 0.9009 |
| Sleep | rs1964463 | 2 | G | 2.840 | 0.500 |  | -0.0080 | 0.0193 | 0.6800 |
| Sleep | rs2394403 | 6 | C | 3.070 | 0.560 |  | 0.0472 | 0.0251 | 0.0600 |
| Sleep | rs4248149 | 6 | T | 3.080 | 0.560 |  | 0.0470 | 0.0251 | 0.0618 |
| Sleep | rs4587207 | 6 | A | 3.140 | 0.560 |  | 0.0472 | 0.0251 | 0.0601 |
| VPA | rs1248860 | 3 | A | 0.051 | 0.007 |  | 0.0104 | 0.0162 | 0.5232 |
| VPA | rs13243553 | 7 | G | 0.039 | 0.007 |  | 0.0146 | 0.0165 | 0.3787 |
| VPA | rs2764261 | 6 | A | 0.030 | 0.005 |  | 0.0110 | 0.0165 | 0.5071 |
| VPA | rs328902 | 7 | T | 0.041 | 0.006 |  | 0.0356 | 0.0175 | 0.0413 |
| VPA | rs3781411 | 10 | C | 0.058 | 0.009 |  | -0.0378 | 0.0242 | 0.1181 |

SNP = single nucleotide polymorphism; Chr = chromosome; EA = effect allele; SE = standard error; MVPA = moderate-to-vigorous physical activity; VPA = vigorous physical activity. *Effect size estimates (β) were obtained from published genome-wide association studies of four lifestyle factors and stroke and represent one unit change in the lifestyle factors per additional copy of the effect allele.

**eTable 4.** **Summary statistics for the genetic variants associated with the lifestyle factors investigated for an association with high density lipoprotein in the present Mendelian randomization study.**

|  |  |  |  | **Lifestyle factor** | |  | **High density lipoprotein result** | | |
| --- | --- | --- | --- | --- | --- | --- | --- | --- | --- |
| **Lifestyle factor** | **SNP** | **Chr** | **EA** | **β** | **SE** |  | **β** | **SE** | **P value** |
| MVPA | rs1043595 | 7 | G | 0.013 | 0.002 |  | -0.0054 | 0.0061 | 0.5803 |
| MVPA | rs1379183 | 7 | A | 0.012 | 0.002 |  | -0.0013 | 0.0049 | 0.5006 |
| MVPA | rs149943 | 6 | G | 0.016 | 0.003 |  | 0.0127 | 0.0074 | 0.0743 |
| MVPA | rs2035562 | 3 | G | 0.014 | 0.002 |  | 0.0044 | 0.0050 | 0.6970 |
| MVPA | rs3094622 | 6 | A | 0.018 | 0.003 |  | 0.0234 | 0.0059 | 0.0006 |
| MVPA | rs7804463 | 7 | T | 0.013 | 0.002 |  | -0.0014 | 0.0048 | 0.8322 |
| MVPA | rs7854466 | 9 | G | 0.014 | 0.002 |  | 0.0025 | 0.0048 | 0.8773 |
| Sedentary | rs25966 | 5 | G | 0.028 | 0.005 |  | -0.0014 | 0.0048 | 0.9594 |
| Sedentary | rs26579 | 5 | G | 0.028 | 0.005 |  | -0.0013 | 0.0051 | 0.8333 |
| Sedentary | rs6801032 | 3 | A | 0.031 | 0.005 |  | 0.0036 | 0.0055 | 0.7027 |
| Sedentary | rs7779206 | 7 | A | 0.028 | 0.005 |  | -0.0034 | 0.0048 | 0.3781 |
| Sleep | rs1191685 | 2 | C | 2.870 | 0.470 |  | 0.0028 | 0.0054 | 0.7015 |
| Sleep | rs1807282 | 2 | T | 2.890 | 0.490 |  | 0.0018 | 0.0058 | 0.7852 |
| Sleep | rs1823125 | 2 | G | 3.010 | 0.500 |  | 0.0040 | 0.0059 | 0.5119 |
| Sleep | rs1964463 | 2 | G | 2.840 | 0.500 |  | 0.0028 | 0.0058 | 0.4954 |
| Sleep | rs2394403 | 6 | C | 3.070 | 0.560 |  | -0.0079 | 0.0066 | 0.2064 |
| Sleep | rs4248149 | 6 | T | 3.080 | 0.560 |  | -0.0086 | 0.0049 | 0.0821 |
| Sleep | rs4587207 | 6 | A | 3.140 | 0.560 |  | -0.0085 | 0.0066 | 0.1639 |
| VPA | rs1248860 | 3 | A | 0.051 | 0.007 |  | 0.0069 | 0.0048 | 0.3967 |
| VPA | rs13243553 | 7 | G | 0.039 | 0.007 |  | -0.0030 | 0.0051 | 0.8170 |
| VPA | rs328902 | 7 | T | 0.041 | 0.006 |  | 0.0024 | 0.0051 | 0.3995 |
| VPA | rs3781411 | 10 | C | 0.058 | 0.009 |  | -0.0011 | 0.0048 | 0.7896 |

SNP = single nucleotide polymorphism; Chr = chromosome; EA = effect allele; SE = standard error; MVPA = moderate-to-vigorous physical activity; VPA = vigorous physical activity. *Effect size estimates (β) were obtained from published genome-wide association studies of four lifestyle factors and stroke and represent one unit change in the lifestyle factors per additional copy of the effect allele.

**eTable 5.** **Summary statistics for the genetic variants associated with the lifestyle factors investigated for an association with low density lipoprotein in the present Mendelian randomization study.**

|  |  |  |  | **Lifestyle factor** | |  | **Low density lipoprotein result** | | |
| --- | --- | --- | --- | --- | --- | --- | --- | --- | --- |
| **Lifestyle factor** | **SNP** | **Chr** | **EA** | **β** | **SE** |  | **β** | **SE** | **P value** |
| MVPA | rs1043595 | 7 | G | 0.013 | 0.002 |  | -0.0036 | 0.0066 | 0.5198 |
| MVPA | rs1379183 | 7 | A | 0.012 | 0.002 |  | 0.0016 | 0.0053 | 0.8953 |
| MVPA | rs149943 | 6 | G | 0.016 | 0.003 |  | -0.0020 | 0.0083 | 0.5593 |
| MVPA | rs2035562 | 3 | G | 0.014 | 0.002 |  | 0.0000 | 0.0054 | 0.9325 |
| MVPA | rs3094622 | 6 | A | 0.018 | 0.003 |  | 0.0099 | 0.0063 | 0.1636 |
| MVPA | rs7804463 | 7 | T | 0.013 | 0.002 |  | -0.0099 | 0.0052 | 0.1124 |
| MVPA | rs7854466 | 9 | G | 0.014 | 0.002 |  | 0.0053 | 0.0053 | 0.1899 |
| Sedentary | rs25966 | 5 | G | 0.028 | 0.005 |  | 0.0046 | 0.0053 | 0.1856 |
| Sedentary | rs26579 | 5 | G | 0.028 | 0.005 |  | 0.0008 | 0.0055 | 0.7172 |
| Sedentary | rs6801032 | 3 | A | 0.031 | 0.005 |  | 0.0051 | 0.0060 | 0.5815 |
| Sedentary | rs7779206 | 7 | A | 0.028 | 0.005 |  | -0.0075 | 0.0052 | 0.1504 |
| Sleep | rs1191685 | 2 | C | 2.870 | 0.470 |  | 0.0132 | 0.0059 | 0.0616 |
| Sleep | rs1807282 | 2 | T | 2.890 | 0.490 |  | 0.0119 | 0.0063 | 0.0388 |
| Sleep | rs1823125 | 2 | G | 3.010 | 0.500 |  | 0.0100 | 0.0064 | 0.0813 |
| Sleep | rs1964463 | 2 | G | 2.840 | 0.500 |  | 0.0015 | 0.0063 | 0.7336 |
| Sleep | rs2394403 | 6 | C | 3.070 | 0.560 |  | -0.0163 | 0.0071 | 0.0222 |
| Sleep | rs4248149 | 6 | T | 3.080 | 0.560 |  | -0.0107 | 0.0052 | 0.0387 |
| Sleep | rs4587207 | 6 | A | 3.140 | 0.560 |  | -0.0162 | 0.0071 | 0.0247 |
| VPA | rs1248860 | 3 | A | 0.051 | 0.007 |  | 0.0013 | 0.0052 | 0.7614 |
| VPA | rs13243553 | 7 | G | 0.039 | 0.007 |  | -0.0093 | 0.0056 | 0.1656 |
| VPA | rs328902 | 7 | T | 0.041 | 0.006 |  | 0.0026 | 0.0055 | 0.9819 |
| VPA | rs3781411 | 10 | C | 0.058 | 0.009 |  | -0.0047 | 0.0052 | 0.4161 |

SNP = single nucleotide polymorphism; Chr = chromosome; EA = effect allele; SE = standard error; MVPA = moderate-to-vigorous physical activity; VPA = vigorous physical activity. *Effect size estimates (β) were obtained from published genome-wide association studies of four lifestyle factors and stroke and represent one unit change in the lifestyle factors per additional copy of the effect allele.

**eTable 6.** **Summary statistics for the genetic variants associated with the lifestyle factors investigated for an association with total cholesterol in the present Mendelian randomization study.**

|  |  |  |  | **Lifestyle factor** | |  | **Total cholesterol result** | | |
| --- | --- | --- | --- | --- | --- | --- | --- | --- | --- |
| **Lifestyle factor** | **SNP** | **Chr** | **EA** | **β** | **SE** |  | **β** | **SE** | **P value** |
| MVPA | rs1043595 | 7 | G | 0.013 | 0.002 |  | -0.0055 | 0.0065 | 0.4564 |
| MVPA | rs1379183 | 7 | A | 0.012 | 0.002 |  | -0.0010 | 0.0052 | 0.5514 |
| MVPA | rs149943 | 6 | G | 0.016 | 0.003 |  | 0.0095 | 0.0081 | 0.3282 |
| MVPA | rs2035562 | 3 | G | 0.014 | 0.002 |  | -0.0032 | 0.0053 | 0.7114 |
| MVPA | rs3094622 | 6 | A | 0.018 | 0.003 |  | 0.0302 | 0.0062 | 5.571×10^-6^ |
| MVPA | rs7804463 | 7 | T | 0.013 | 0.002 |  | -0.0103 | 0.0051 | 0.1026 |
| MVPA | rs7854466 | 9 | G | 0.014 | 0.002 |  | 0.0062 | 0.0051 | 0.1889 |
| Sedentary | rs25966 | 5 | G | 0.028 | 0.005 |  | 0.0045 | 0.0052 | 0.3077 |
| Sedentary | rs26579 | 5 | G | 0.028 | 0.005 |  | 0.0023 | 0.0054 | 0.6141 |
| Sedentary | rs6801032 | 3 | A | 0.031 | 0.005 |  | 0.0036 | 0.0059 | 0.6834 |
| Sedentary | rs7779206 | 7 | A | 0.028 | 0.005 |  | -0.0067 | 0.0051 | 0.2771 |
| Sleep | rs1191685 | 2 | C | 2.870 | 0.470 |  | 0.0103 | 0.0057 | 0.1108 |
| Sleep | rs1807282 | 2 | T | 2.890 | 0.490 |  | 0.0120 | 0.0062 | 0.0388 |
| Sleep | rs1823125 | 2 | G | 3.010 | 0.500 |  | 0.0098 | 0.0063 | 0.0860 |
| Sleep | rs1964463 | 2 | G | 2.840 | 0.500 |  | 0.0034 | 0.0062 | 0.5780 |
| Sleep | rs2394403 | 6 | C | 3.070 | 0.560 |  | -0.0221 | 0.0070 | 0.0014 |
| Sleep | rs4248149 | 6 | T | 3.080 | 0.560 |  | -0.0135 | 0.0051 | 0.0061 |
| Sleep | rs4587207 | 6 | A | 3.140 | 0.560 |  | -0.0225 | 0.0070 | 0.0014 |
| VPA | rs1248860 | 3 | A | 0.051 | 0.007 |  | 0.0000 | 0.0051 | 0.9101 |
| VPA | rs13243553 | 7 | G | 0.039 | 0.007 |  | -0.0095 | 0.0054 | 0.1337 |
| VPA | rs328902 | 7 | T | 0.041 | 0.006 |  | 0.0014 | 0.0054 | 0.9573 |
| VPA | rs3781411 | 10 | C | 0.058 | 0.009 |  | -0.0055 | 0.0050 | 0.2781 |

SNP = single nucleotide polymorphism; Chr = chromosome; EA = effect allele; SE = standard error; MVPA = moderate-to-vigorous physical activity; VPA = vigorous physical activity. *Effect size estimates (β) were obtained from published genome-wide association studies of four lifestyle factors and stroke and represent one unit change in the lifestyle factors per additional copy of the effect allele.

**eTable 7.** **Summary statistics for the genetic variants associated with the lifestyle factors investigated for an association with triglycerides in the present Mendelian randomization study.**

|  |  |  |  | **Lifestyle factor** | |  | **Triglycerides result** | | |
| --- | --- | --- | --- | --- | --- | --- | --- | --- | --- |
| **Lifestyle factor** | **SNP** | **Chr** | **EA** | **β** | **SE** |  | **β** | **SE** | **P value** |
| MVPA | rs1043595 | 7 | G | 0.013 | 0.002 |  | -0.0031 | 0.006 | 0.2581 |
| MVPA | rs1379183 | 7 | A | 0.012 | 0.002 |  | -0.0052 | 0.0047 | 0.3245 |
| MVPA | rs149943 | 6 | G | 0.016 | 0.003 |  | 0.0131 | 0.0075 | 0.05911 |
| MVPA | rs2035562 | 3 | G | 0.014 | 0.002 |  | -0.0043 | 0.0049 | 0.4569 |
| MVPA | rs3094622 | 6 | A | 0.018 | 0.003 |  | 0.0307 | 0.0058 | 7.182×10^-8^ |
| MVPA | rs7804463 | 7 | T | 0.013 | 0.002 |  | -0.0037 | 0.0047 | 0.1856 |
| MVPA | rs7854466 | 9 | G | 0.014 | 0.002 |  | 0.0034 | 0.0047 | 0.5369 |
| Sedentary | rs25966 | 5 | G | 0.028 | 0.005 |  | -0.0005 | 0.0047 | 0.8687 |
| Sedentary | rs26579 | 5 | G | 0.028 | 0.005 |  | 0.0028 | 0.0049 | 0.5912 |
| Sedentary | rs6801032 | 3 | A | 0.031 | 0.005 |  | 0.003 | 0.0054 | 0.9561 |
| Sedentary | rs7779206 | 7 | A | 0.028 | 0.005 |  | 0.0039 | 0.0047 | 0.5236 |
| Sleep | rs1191685 | 2 | C | 2.870 | 0.470 |  | 0.0024 | 0.0053 | 0.9443 |
| Sleep | rs1807282 | 2 | T | 2.890 | 0.490 |  | 0.0047 | 0.0057 | 0.6247 |
| Sleep | rs1823125 | 2 | G | 3.010 | 0.500 |  | 0.0013 | 0.0058 | 0.9947 |
| Sleep | rs1964463 | 2 | G | 2.840 | 0.500 |  | 0.0007 | 0.0057 | 0.9435 |
| Sleep | rs2394403 | 6 | C | 3.070 | 0.560 |  | -0.015 | 0.0065 | 0.03005 |
| Sleep | rs4248149 | 6 | T | 3.080 | 0.560 |  | -0.0025 | 0.0048 | 0.5791 |
| Sleep | rs4587207 | 6 | A | 3.140 | 0.560 |  | -0.0101 | 0.0065 | 0.1509 |
| VPA | rs1248860 | 3 | A | 0.051 | 0.007 |  | -0.0003 | 0.0047 | 0.6761 |
| VPA | rs13243553 | 7 | G | 0.039 | 0.007 |  | -0.0012 | 0.0049 | 0.5201 |
| VPA | rs328902 | 7 | T | 0.041 | 0.006 |  | -0.0014 | 0.005 | 0.7744 |
| VPA | rs3781411 | 10 | C | 0.058 | 0.009 |  | -0.0041 | 0.0047 | 0.4364 |

SNP = single nucleotide polymorphism; Chr = chromosome; EA = effect allele; SE = standard error; MVPA = moderate-to-vigorous physical activity; VPA = vigorous physical activity. *Effect size estimates (β) were obtained from published genome-wide association studies of four lifestyle factors and stroke and represent one unit change in the lifestyle factors per additional copy of the effect allele.

**eTable 8.** **Associations between lifestyle factors (generally predicted per 1 SD increase of continuous factors or per 1 unit log odds increase of binary factors) and cardiovascular diseases in sensitivity analyses.**

| **Diseases** | **Simple median** | |  | **Weighted median** | |  | **MR-Egger** | |  | **(intercept)** | |
| --- | --- | --- | --- | --- | --- | --- | --- | --- | --- | --- | --- |
|  | **OR (95% CI)** | **P** |  | **OR (95% CI)** | **P** |  | **OR (95% CI)** | **P** |  | **Beta±SE** | **P** |
| **MVPA** |  |  |  |  |  |  |  |  |  |  |  |
| MI | 1.75(0.8,3.84) | 0.163 |  | 1.64(0.74,3.63) | 0.225 |  | 40.69(0.12,13643) | 0.212 |  | -0.047±0.288 | 0.288 |
| CAD | 2.25(0.6,8.47) | 0.229 |  | 1.97(0.58,6.74) | 0.278 |  | 0.03(0,200.14) | 0.444 |  | 0.052±0.061 | 0.399 |
| Stroke | 1.74(0.54,5.61) | 0.351 |  | 1.78(0.59,5.34) | 0.304 |  | 0.42(0.01,14.44) | 0.632 |  | 0.018±0.028 | 0.512 |
| **VPA** |  |  |  |  |  |  |  |  |  |  |  |
| MI | 0.71(0.52,0.97) | 0.031 |  | 0.74(0.56,0.99) | 0.041 |  | 1.01(0.35,2.97) | 0.982 |  | -0.014±0.024 | 0.56 |
| CAD | 0.66(0.44,0.98) | 0.038 |  | 0.67(0.45,0.99) | 0.044 |  | 0.19(0.02,1.61) | 0.129 |  | 0.057±0.051 | 0.258 |
| Stroke | 1.45(0.87,2.43) | 0.158 |  | 1.37(0.86,2.16) | 0.185 |  | 0.34(0.04,2.55) | 0.291 |  | 0.058±0.045 | 0.197 |
| **Sleep duration** | |  |  |  |  |  |  |  |  |  |  |
| MI | 1(1,1.01) | 0.714 |  | 1(1,1.01) | 0.816 |  | 1.07(0.88,1.31) | 0.482 |  | -0.208±0.292 | 0.477 |
| CAD | 1(1,1.01) | 0.677 |  | 1(0.99,1) | 0.55 |  | 1.08(0.95,1.22) | 0.238 |  | -0.233±0.189 | 0.218 |
| Stroke | 1(0.99,1.01) | 0.845 |  | 1(0.99,1.01) | 0.771 |  | 1.22(1.05,1.41) | 0.008 |  | -0.568±0.220 | 0.01 |
| **Sedentary** |  |  |  |  |  |  |  |  |  |  |  |
| MI | 1.32(0.83,2.1) | 0.24 |  | 1.33(0.83,2.11) | 0.233 |  | 2.7(0,3471162.12) | 0.89 |  | -0.018±0.206 | 0.931 |
| CAD | 1.27(0.68,2.38) | 0.449 |  | 1.2(0.64,2.24) | 0.563 |  | 0.01(0,217509.98) | 0.617 |  | 0.125±0.241 | 0.606 |
| Stroke | 0.85(0.44,1.63) | 0.618 |  | 0.86(0.45,1.65) | 0.647 |  | 0.04(0,31697.72) | 0.644 |  | 0.087±0.198 | 0.66 |

MVPA = moderate-to-vigorous physical activity, VPA = vigorous physical activity; MI = myocardial infarction, CAD = coronary artery disease.

**eTable 9.** **Associations between lifestyle factors (generally predicted per 1 SD increase of continuous factors or per 1 unit log odds increase of binary factors) and lipids in sensitivity analyses.**

| **Lipids** | **Simple median** | |  | **Weighted median** | |  | **MR-Egger** | |  | **(intercept)** | |
| --- | --- | --- | --- | --- | --- | --- | --- | --- | --- | --- | --- |
|  | **Beta±SE** | **P** |  | **Beta±SE** | **P** |  | **Beta±SE** | **P** |  | **Beta±SE** | **P** |
| **MVPA** |  |  |  |  |  |  |  |  |  |  |  |
| HDL | 0.179±0.223 | 0.423 |  | 0.211±0.217 | 0.33 |  | 4.516±1.14 | 0.000 |  | -0.059±0.016 | 0.000 |
| LDL | 0±0.231 | 1.000 |  | 0.072±0.211 | 0.732 |  | 1.921±1.239 | 0.121 |  | -0.027±0.017 | 0.125 |
| TC | -0.083±0.259 | 0.747 |  | 0.014±0.238 | 0.951 |  | 5.873±1.422 | 0.000 |  | -0.08±0.02 | 0.000 |
| TG | -0.238±0.237 | 0.315 |  | -0.213±0.22 | 0.333 |  | 6.075±1.118 | 0.000 |  | -0.082±0.016 | 0.000 |
| **VPA** |  |  |  |  |  |  |  |  |  |  |  |
| HDL | 0.02±0.064 | 0.755 |  | 0.025±0.064 | 0.697 |  | 0.088±0.339 | 0.794 |  | -0.003±0.016 | 0.863 |
| LDL | -0.028±0.066 | 0.674 |  | -0.032±0.067 | 0.64 |  | 0.038±0.423 | 0.928 |  | -0.004±0.021 | 0.836 |
| TC | -0.047±0.064 | 0.461 |  | -0.052±0.066 | 0.425 |  | 0.025±0.385 | 0.949 |  | -0.005±0.019 | 0.807 |
| TG | -0.032±0.059 | 0.58 |  | -0.033±0.058 | 0.566 |  | -0.112±0.308 | 0.716 |  | 0.004±0.015 | 0.811 |
| **Sleep duration** | |  |  |  |  |  |  |  |  |  |  |
| HDL | 0.001±0.001 | 0.561 |  | 0±0.001 | 0.806 |  | -0.043±0.02 | 0.031 |  | 0.128±0.06 | 0.034 |
| LDL | 0.001±0.002 | 0.751 |  | 0±0.001 | 0.968 |  | -0.089±0.03 | 0.003 |  | 0.264±0.088 | 0.003 |
| TC | 0.001±0.002 | 0.468 |  | 0.001±0.001 | 0.662 |  | -0.107±0.032 | 0.001 |  | 0.315±0.095 | 0.001 |
| TG | 0±0.001 | 0.806 |  | 0±0.001 | 0.861 |  | -0.041±0.02 | 0.037 |  | 0.121±0.059 | 0.04 |
| **Sedentary** |  |  |  |  |  |  |  |  |  |  |  |
| HDL | -0.048±0.1 | 0.629 |  | -0.048±0.1 | 0.627 |  | 1.887±2.061 | 0.36 |  | -0.055±0.059 | 0.352 |
| LDL | 0.096±0.116 | 0.407 |  | 0.093±0.116 | 0.422 |  | 1.975±2.651 | 0.456 |  | -0.056±0.076 | 0.46 |
| TC | 0.099±0.113 | 0.382 |  | 0.099±0.113 | 0.383 |  | 1.237±2.545 | 0.627 |  | -0.035±0.073 | 0.634 |
| TG | 0.098±0.098 | 0.314 |  | 0.098±0.098 | 0.313 |  | 0.318±2.02 | 0.875 |  | -0.007±0.058 | 0.906 |

HDL = high density lipoprotein, LDL = low density lipoprotein, TC = total cholesterol, TG = triglycerides.
